# MADS-Box Protein Complex VvAG2, VvSEP3 and VvAGL11 Regulates the Formation of Ovules in *Vitis vinifera* L. cv. ‘Xiangfei’

**DOI:** 10.3390/genes12050647

**Published:** 2021-04-26

**Authors:** Yan Wang, Zhenhua Liu, Jiang Wu, Liang Hong, Jinjun Liang, Yangmei Ren, Pingyin Guan, Jianfang Hu

**Affiliations:** College of Horticulture, China Agricultural University, Beijing 100193, China; tracywang0104@163.com (Y.W.); liuzhenhua93@163.com (Z.L.); wj619432057@163.com (J.W.); hl1006436066@163.com (L.H.); liangjinjun1989@163.com (J.L.); yangmei_ren@163.com (Y.R.); pyguan@cau.edu.cn (P.G.)

**Keywords:** grape, ovule formation, MADS-box genes, *VvAG2*, transgenic tomato, tetramerization

## Abstract

The phenomenon of multi-carpel and multi-ovule exists in the grapevine cultivar ‘Xiangfei’, but the mechanism of ovule formation is seldom reported. In this study, we observed the ovule formation process by using ‘Xiangfei’ grapes. The role of the *VvAG2* (*VvAGAMOUS*) gene in ovule formation was identified, and we explored the relationship between VvAG2, VvSEP3(VvMADS4) and VvAGL11(VvMADS5) proteins. The results showed that the ovule primordium appeared when the inflorescence length of ‘Xiangfei’ grapes were 4–5 cm long; the relative expression levels of *VvAG2*, *VvAGL11* and *VvSEP3* genes were higher during ovule formation, and the expression levels of *VvAG2* gene was the highest. Transgenic tomato (*Solanum lycopersicum*) plants expressing *VvAG2* produced higher numbers of ovules and carpels than the wild type. Moreover, yeast two-hybrid and yeast three-hybrid experiments demonstrated that VvSEP3 acts as a bridge and interacts with VvAG2 and VvAGL11 proteins, respectively. Meanwhile, a homodimer can be formed between VvSEP3 and VvSEP3, but there was no interaction between VvAG2 and VvAGL11. These findings suggest that the *VvAG2* gene is involved in the formation of ovules, and VvAG2/VvSEP3 together with VvAGL11/VvSEP3 can form a tetrameric complex. In summary, our data showed that *VvAG2* along with *VvSEP3* and *VvAGL11* jointly regulate the ovule formation of ‘Xiangfei’ grapes.

## 1. Introduction

As the site of double fertilization in angiosperms, ovules are also the precursor of seeds. The efficient formation and development of numerous ovules are essential for the sexual reproduction and progeny reproduction of plants [1,2]. Ovule development takes place inside the gynoecium, and the ovule primordium is initiated by periclinal divisions in the subepidermal tissue of the placenta [3]. The ovule has a complex morphological structure and is composed of the funiculus, integument, micropyle and nucellus. The funiculus not only supports the ovule but also plays a role in transporting nutrients. The integument is usually divided into two protective layers—the inner integument and outer integument—which can protect the nucellus tissue and eventually develop into the seed coat. As the guidance and channel for the male gametophyte sperm entering the embryo sac, the micropyle is important for double fertilization. For the nucellus, it plays a crucial role in determining the seed structure and fertilization products, and is an important site for the formation of megasporocytes and female gametophytes [4]. Although ovules are found in most plants, their formation and morphology vary greatly among different varieties.

Ovule formation was studied at the morphological, genetic, and molecular levels in the model plant *Arabidopsis thaliana* [3,5]. Several genes, which are associated with the development of floral organs, have been identified to participate in ovule formation in the last decades. For instance, the C-class gene *AGAMOUS* (*AG*) is reported to regulate the formation of ovule primordial, and less normal ovules are detected in the *ag bel1* mutant [6,7,8,9]. The D-class genes *SEEDSTICK* (*STK*) and *SHATTERPRO1/2* (*SHP1/SHP2*) also modulate the formation of ovules. Overexpression of the *STK* gene leads to ectopic ovule formation [9,10]. The E-class gene *SEPALLATA* (*SEP*) is also necessary for ovule development. In the *sep1 sep2 sep3* triple mutant, four petals and six stamens of the normal flower are replaced by sepaloid organs, and the carpels are replaced by another abnormal flower which has a similar phenotype [10,11,12]. Therefore, these findings suggest that the characteristic genes of different types of floral organs are involved in the formation of ovules. However, the molecular mechanism of ovule formation in most plants is still unclear.

Most of these genes involved in ovule formation belong to the MADS-box (‘MADS’ is an acronym for four transcription factors: MINICHROMOSOME MAINTENANCE FACTOR1 (MCM1; *Saccharomyces cerevisiae*), AGAMOUS (AG; *Arabidopsis thaliana*), DEFICIENS (DEF; *Antirrhinum majus*) and SERUM RESPONSE FACTOR (SRF; *Homo sapiens*)) transcription factor family. The identities of different types of floral organs are specified by homeotic MADS transcription factors that interact in a combinatorial fashion. Two MADS-box proteins interact to form a dimer, and the C-terminus of the two dimers can be further linked to form a tetramer protein. One tetramer can bind to a target gene promoter with two CArG-box (A class of DNA sequence motif combined with MADS-box proteins, having a consistent 5’-CC(A /T)_6_G-3’ sequence or similar sequences) cis-acting elements, which initiates the expression of the target gene and determines the characteristics of each organ whorl [13]. The formation of higher-order complexes is a necessary molecular mechanism for plant MADS-box proteins to perform their functions; MADS-box family proteins’ SEPs play a key role in the multimerization process of this higher-order complex [11,14,15]. In Arabidopsis, it was confirmed that AGAMOUS (AG), SEEDSTICK (STK), and SHATTERPROOF1/2 (SHP1/2) formed a protein complex under the bridge action of SEP3 and participated in ovule development [10]. In addition, BELL1 (BEL1) can form a complex with the members of the MADS-box family, AG and SEPALLATA3 (SEP3), and this complex is involved in the development of the ovule primordium of Arabidopsis [8]. Although research on the tetramer model of floral organ formation and development in model plants has been well studied, it has not been reported whether there are tetramers involved in regulation during grape ovule formation.

The ovule of grape (*Vitis vinifera*) belongs to the anatrophic ovule type, which starts from the placental meristem inside the carpel, that is, the ovule primordium. Most varieties of grape have two carpels and four ovules [16,17]. However, a few varieties developed multi-carpel variation during evolution [18], and the number of ovules increases correspondingly. In past decades, this topic has been deeply studied and verified in the model plant tomato: increasing the number of carpels helps to greatly increase the tomato fruit size during fruit domestication [19,20]. The tomato *AGAMOUS*(*TAG1*) has been identified as the key regulator gene in carpel development [21]. At present, most of the research related to the grape ovule focuses on seed abortion after ovule formation. *VvAGL11* has been proposed as the major candidate gene involved in *Vitis vinifera* seed morphogenesis, and its expression is responsible for the erroneous development of a highly essential integument layer [22]. Rahman et al. characterize the molecular level of *VroAGL11* in *Vitis rotundifolia* and analyze its divergence from other plants, suggesting that the *VroAGL11* gene controls the seed morphogenesis after ovule formation in muscadine grapes [23]. The formation of the ovule is the premise of seed forming. What kind of regulatory mechanism exists in the process of grape ovule formation?

In this study, we demonstrated that the *VvAG2* gene along with *VvSEP3* and *VvAGL11* is involved in the ovule formation of ‘Xiangfei’ grapes. The formations of ovules and other floral organ primordia in the ‘Xiangfei’ grape have been elucidated. The heterologous overexpression of *VvAG2* in the tomato increased the number of carpels and ovules of these genetically modified tomato plants. In addition, we proved that the VvAG2, VvSEP3 and VvAGL11 proteins of the MADS-box family might have participated in the formation of ‘Xiangfei’ grape ovules in the form of tetramers.

## 2. Materials and Methods

### 2.1. Plant Materials

**Figure 1:**
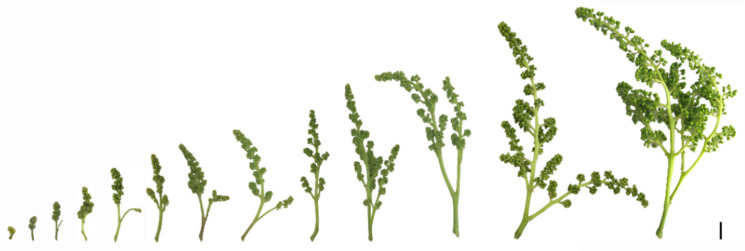
The lengths of ‘Xiangfei’ grape inflorescences used for sampling. From left to right: <1, 1–2, 2–3, 3–4, 4–5, 5–6, 6–7, 7–8, 8–9, 9–10, 10–12, 12–14, 14–16 cm, corresponding sampling time: 40, 34, 32, 30, 28, 26, 24, 22, 20, 18, 14, 10, 6 DBF (Day before anthesis). Scale bar = 1 cm.

The *V. vinifera* cultivar ‘Xiangfei’ was planted in the grapevine nursery in Beijing Wenquan town, China. Eleven-year-old ‘Xiangfei’ grapes were harvested as the experimental materials between 2018 and 2019. Inflorescences of different lengths at different developmental stages (<1, 1–2, 2–3, 3–4, 4–5, 5–6, 6–8, 8–9, 9–10, 10–12, 12–14 and 14–16 cm) were collected for organizational structure observation and RNA extraction (Figure 1). The florets were harvested at the anthesis stage, and their carpel numbers and the number of ovules in different locules were determined using a stereo microscope. There is no allelic variation in the sampled biological material. The ‘Xiangfei’ grape was bred by crossbreeding at the Beijing Academy of Agriculture and Forestry Sciences, China, in 1982. Its parents were ‘Cardinal’ and ‘73-7-6’ (‘Muscat Hamburg’ × ’Pearl of Csaba’).

The Micro-Tom tomato seeds (PanAmerican Seed Company, Chicago, IL, USA) were used for the genetic transformations.

### 2.2. Organizational Structure Observation

The fresh inflorescences of different lengths were collected and used to make paraffin slices for organizational structure observation. After being fixed in FAA solid solution (50% (*v*/*v*) ethanol, 5% (*v*/*v*) glacial acetic acid and 5% (*v*/*v*) formaldehyde) for at least 24 h, the samples were dehydrated with ethanol aqueous solutions of different concentration gradients. The samples were then placed in a mixture of different proportions of ethanol and n-butanol (2 h per step). The samples were then dipped in wax before finally being embedded in paraffin wax. Using a paraffin slicing machine, the wax block was cut into 9-μm wax strips and placed on glass slides. Tissue dyeing was performed by the method of Safranin O-Fast Green (Sigma-Aldrich, Saint louis, MO, USA). The prepared paraffin sections were observed and photographed using an Olympus CX31 microscope (Olympus Corporation, Tokyo, Japan). The processes of dehydration and transparency were carried out according to the method described by Liang et al. [18].

In order to observe and count the number of the Micro-Tom tomato ovules, small fruits with a transverse diameter of 4–6 mm were harvested one week after anthesis, and then the number of ovules was observed and counted using a stereo microscope.

### 2.3. RNA Extraction and qRT-PCR

To extract the total RNA from the collected grape inflorescences, the CTAB method was used [24]. The RNA quality was measured with the atomic ultraviolet spectrophotometer (OD260/280 = 1.8–2.0). The cDNA synthesis was initiated from 2 µg total RNA using a reverse transcription kit (Promega, Madison, WI, USA), and the procedure was as follows: the samples were incubated for 5 min at 70 °C and 1 h at 42 °C, and then placed on ice immediately. The qRT–PCR analyses were conducted on an ABI PRISM 7500 system (Thermo Fisher Scientific, Waltham, MA, USA) as described previously in Liu et al. [25]. The reaction volume of qRT–PCR was 10 μL 2× Bestar SybrGreen qPCR Mastermix (DBI, Ludwigshafen, Germany) + 6 μL double distilled water (ddH2O) + 2 μL sample cDNA (0.025 μg) + 0.8 μL forward primers + 0.8 μL reverse primer + 0.4 μL 50× ROX Reference Dye. Three biological replicates and two technical replicates were performed. The *UBQ* gene was used as a reference gene. The relative gene expression level was calculated using the 2^−ΔΔCt^ method [26].

### 2.4. In Situ Hybridization

An in situ hybridization analysis was performed to detect the spatiotemporal distribution of VvAG2, VvSEP3, and VvAGL11 on ‘Xiangfei’ grape inflorescences of 4–5 cm long. The collected material was fixed in paraformaldehyde, then the samples were dehydrated and transparent, and then embedded in paraffin wax. The probes for in situ hybridization were synthesized by PCR approach, using the ‘Xiangfei’ grape cDNA as templates. The probe sequence lengths of VvAG2, VvSEP3 and VvAGL11 were 179 bp, 199 bp and 257 bp, respectively. The PCR products were ligated into the pMD-19T vector (Takara Bio, Kusatsu City, Japan) digested with *EcoRI* and *HindIII* for sequencing (>99% similarity). The obtained PCR products were cloned into the pSPT-18 vector (Roche, Shanghai, China). The antisense probes were synthesized using a DIG RNA labeling kit (Sp6/T7; Roche). The in situ hybridization was conducted as described by Drews et al. [27].

### 2.5. Genetic Modification

The full-length *VvAG2* cDNA sequence was amplified by the PCR approach, using ‘Xiangfei’ grape cDNA as templates. The PCR products were cloned into the pCAMBIA 1305.1 plant expression vector to generate the *35S::GFP-VvAG2* transgene. The *35S::GFP-VvAG2* transgene was then transferred into *Agrobacterium tumefaciens* EHA105 cells using the freeze–thaw method [28].

The transgenic Agrobacterium cells were used to infect the cut tomato cotyledons (15 day old) for 10 min by the leaf disk method. After the infected cotyledons were absorbed by filter paper to remove the residual bacterial liquid, they were placed in the preculture medium (M1: Murashige and Skoog 21 + 20 g/L sucrose + 5 g/L agar + 2.0 mg/L zeatin riboside (ZR) + 0.5 mg/L indole-3-acetic acid (IAA)) under dark conditions for 36 h. Then the cotyledons were transferred to the screening medium (M2: MS + 20 g/L sucrose + 5 g/L agar + 2.0 mg/L ZR + 0.5 mg/L IAA + 500 mg/L Cef + 10 mg/L Hygromix B) for 16 h light/8 h dark under the light intensity of 2000–3000 lx. The transgenic plants were cultured in an artificial climate incubator HPG-280 HX (HDL, Changzhou City, China) with the 16 h light/8 h dark photoperiod. The transgenic methods were described as Liang et al. [18]. The wild-type Micro-Tom tomato was used as the negative control for the tomato transformation.

### 2.6. Subcellular Localization

To get the transgenes of *35S::GFP-VvAG2*, *35S::GFP-VvSEP3* and *35S::GFP-VvAGL11*, the full-length cDNA sequences of *VvAG2*, *VvSEP3* and *VvAGL11* were cloned into pCAMBIA 1305.1 plant constitutive expression vectors, respectively. These constructed vectors were independently transferred into *Agrobacterium* EHA105 cells using the freeze–thaw method [28]. *Agrobacterium* was activated by oscillating culture to an OD value of 0.6–1.0. After centrifugation, the resulting *Agrobacterium* precipitates were resuspended with a solution (1 mM MgCl_2_, 1 mM MES-KOH and 50 μM acetosyringone) as an infection solution. The infection solution was injected into 4- to 6-week-old *Nicotiana benthamiana* leaves with a 1 mL syringe without a needle for transient expression. The fluorescence signals in the transgenic leaves were observed under confocal microscopy after 48 h of cultivation.

### 2.7. Yeast Two-Hybrid (Y2H) Assay

The full-length CDSs (Coding sequence) of *VvAG2*, *VvSEP3* and *VvAGL11* were independently cloned into pGADT7 and pGBKT7 (Vectors for yeast two-hybrid). All constructs were confirmed by sequencing, and then transferred into yeast strain AH109. Protein interactions were analyzed on the yeast four-deficiency solid (SD/-Trp/-Leu/-Ade/-His) medium. The positive hybridization colonies were detected by X-α-Gal. The combination of pGADT7-T (SV40 large T-antigen) and pGBKT7-53 (p53) was used as the positive control.

### 2.8. Yeast Three-Hybrid (Y3H) Assay

The pBridge vector was used to express both the VvSEP3 and VvAGL11. VvSEP3 was ligated into the *NotI-* and *BglⅡ*-digested pBridge vector promoted by Met25 promoter, and the VvAGL11 was ligated into the *EcoRI-* and *BamhI*-digested pBridge vector promoted by ADH1 promoter. The *VvAGL11* gene was linked to the BD in the pBridge vector. The pGADT7 vector was used to express the VvAG2; VvAG2 was ligated into the *NdeI*- and *XhoI*-digested pGADT7 vector. Pairs of constructs were transformed into the AH109 yeast strain. Protein interactions were analyzed on a selective medium lacking Leu, Trp, and His. The positive hybridization colonies were detected by X-α-Gal.

### 2.9. Bimolecular Fluorescence Complementation (BiFC) Assay

The full-length CDS without stop condons of *VvAG2*, *VvSEP3* and *VvAGL11* were cloned and inserted into the pSPYNE and pSPYCE vectors, with each of them containing half of YFP (N- or C-terminus) to generate the fusion proteins [29]. After *Agrobacterium* transformation and co-injection of *Nicotiana* benthamiana, co-expression studies were performed in the abaxial sides of tobacco leaves (4−6 weeks old). The fluorescence of the expressed fusion proteins was detected after 48 h of infiltration, and fluorescence images were obtained using a confocal microscope. 

The Primer Software 5.0 (Premier Biosoft International, Palo Alto, CA, USA) was used to design the primers for the genes. All primers involved in the experiments are listed in Supplementary Table S1. The sequence names and the accession numbers of all the genes involved in this study are provided in Supplementary Table S2.

## 3. Results

### 3.1. The Number of Ovules Varies Between Different Florets of ‘Xiangfei’ Grape

**Figure 2:**
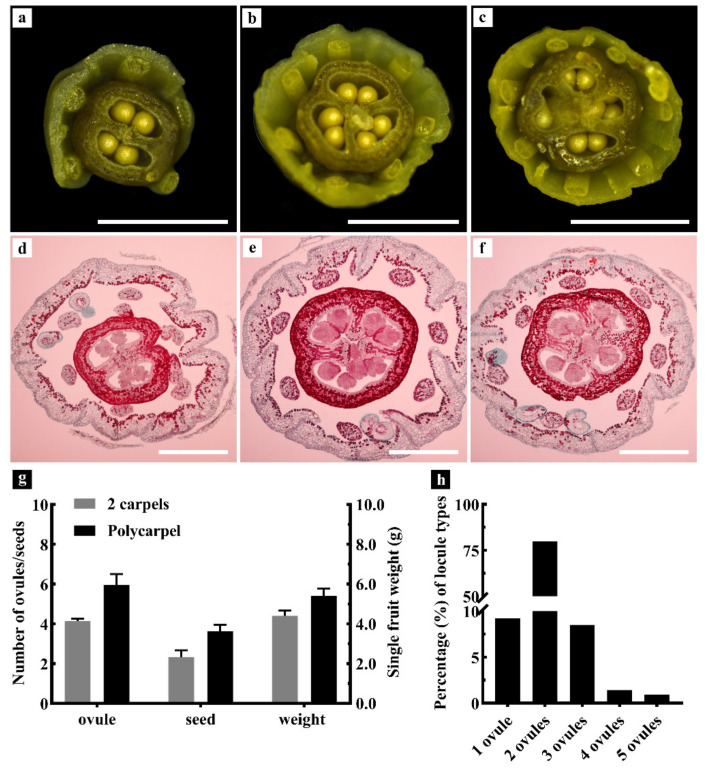
Observation and quantification of the ovule numbers of ‘Xiangfei’ grapes. (**a**–**c**) Ovules in 2, 3, and 4 carpellate florets on the inflorescence (one week before anthesis) were examined under a stereoscopic microscope, scale bars = 1 mm. (**d**–**f**) Paraffin slices. Ovules in 2, 3, and 4 carpellate florets on inflorescences of 8–12 cm long, scale bars = 100 μm. (**g**) The number of ovules in 2-carpel and polycarpel florets, average fruit weight and number of seeds in 2-carpel and polycarpel fruits. Error bars indicate standard errors. (**h**) The proportions of locules with different ovule numbers (*n* > 1000, ‘*n*’ represents the number of locules that were counted).

To observe and count the number of ovules in the ovary of the ‘Xiangfei’ grape, the stereoscope and paraffin section staining were used in this study. We found that most 2-carpel ovaries had 4 ovules (Figure 2a,d). Accordingly, 3-carpel and 4-carpel ovaries generally had 6 and 8 ovules, respectively (Figure 2b,c,f). Sometimes, one locule contained more than 2 ovules (Figure 2e). A statistical analysis revealed that the average number of ovules and seeds in the 2-carpel mature fruits were 4.13 and 2.48, respectively. Further, the single fruit weight was about 4.56 g (Figure 2g). For multi-carpel grapes, the average number of ovules in the ovary was 6.13; the average number of seeds and the single fruit weight in mature fruits were respectively 3.80 and 5.61 g (Figure 2g). Thus, our results revealed that most locules generally contain 2 ovules while several locules had only one ovule or 3–5 ovules (Figure 2h).

### 3.2. Observation on the Ovule Formation Process of ‘Xiangfei’ Grape

**Figure 3:**
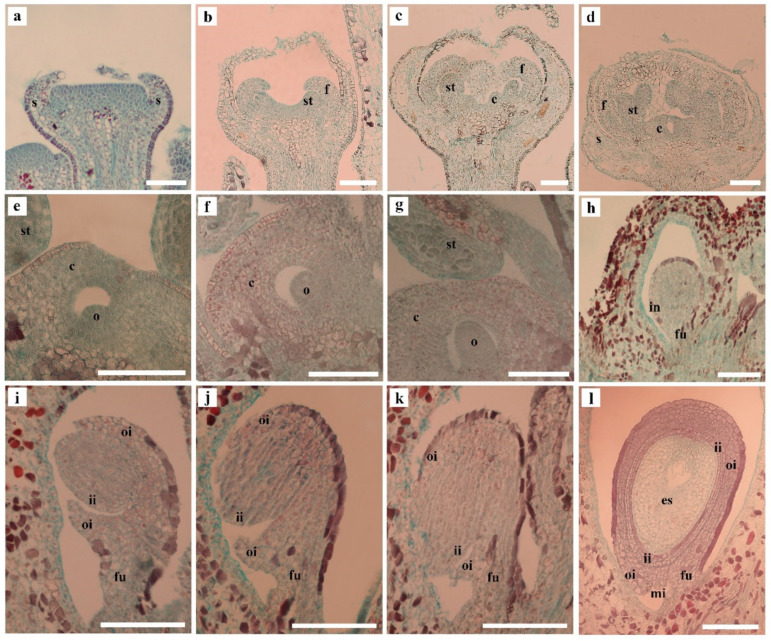
Anatomical structure of ‘Xiangfei’ grape florets in inflorescences at different developmental stages using Safranin O-Fast Green staining. (**a**) Inflorescence length (IL) <1 cm, sepal primordium formed and developed. (**b**) IL: 1–2 cm, flower cap primordium developed, stamen primordium protruded. (**c**) IL: 2–3 cm, carpel primordium formed. (**d**) IL: 3–4 cm, carpel primordium fused. (**e**) IL: 4–5 cm, ovule primordia formed. (**f**–**h**) IL: 5–8 cm, the development of ovule primordium. (**i**–**k**) IL: 8–12 cm, the inversion stage of ovules. (**l**) IL: 12–16 cm, the embryo sac began to develop. Scale bars = 200 μm. o, ovule primordia; s, sepal primordia; f, flower cap primordia; st, stamen primordia; c, carpel primordia; in, integument; oi, outer integument; ii, inner integument; mi, micropyle; es, embryo sac.

Due to the differences in the locule numbers, there are also some differences in the ovules, seeds and single fruit weight of the ‘Xiangfei’ grape. The number of ovules formed in one locule also varies. The ovule formation process of the ‘Xiangfei’ grape was observed using inflorescences of different lengths. When the inflorescence was shorter than 1 cm, the sepal primordium formed and started to develop (Figure 3a). In the length of 1–2 cm, the flower cap primordium was in the developmental stage, and the stamen primordium began to protrude at the edge of the growing point (Figure 3b). The primordia of carpels began to form in the 2–3 cm inflorescences; meanwhile, the stamens started to differentiate into the sporogenous cells (Figure 3c). The carpel primordium continued to grow and began to fuse in the 3–4 cm long inflorescences (Figure 3d). The ovule primordia began to form when the inflorescences were 4–5 cm long (Figure 3e). As the inflorescences grow to 5–8 cm long, the protuberance of the ovule primordium continued to enlarge (Figure 3f,g) and gradually formed the funiculus and integuments (Figure 3h). The ovule entered the inversion stage in the florets of the 8–9 cm inflorescences (Figure 3i). Afterwards, the ovules gradually completed the inversion in the florets of the 8–12 cm inflorescences, and the nucleus tissue was surrounded by the inner and outer integuments (Figure 3i–k). With further inflorescence growth, the embryo sac began to develop in the florets of the 12–16 cm inflorescences (Figure 3l) and gradually matured.

### 3.3. The C-Class, D-Class and E-Class Genes Differentially Expressed during Ovule Formation

**Figure 4:**
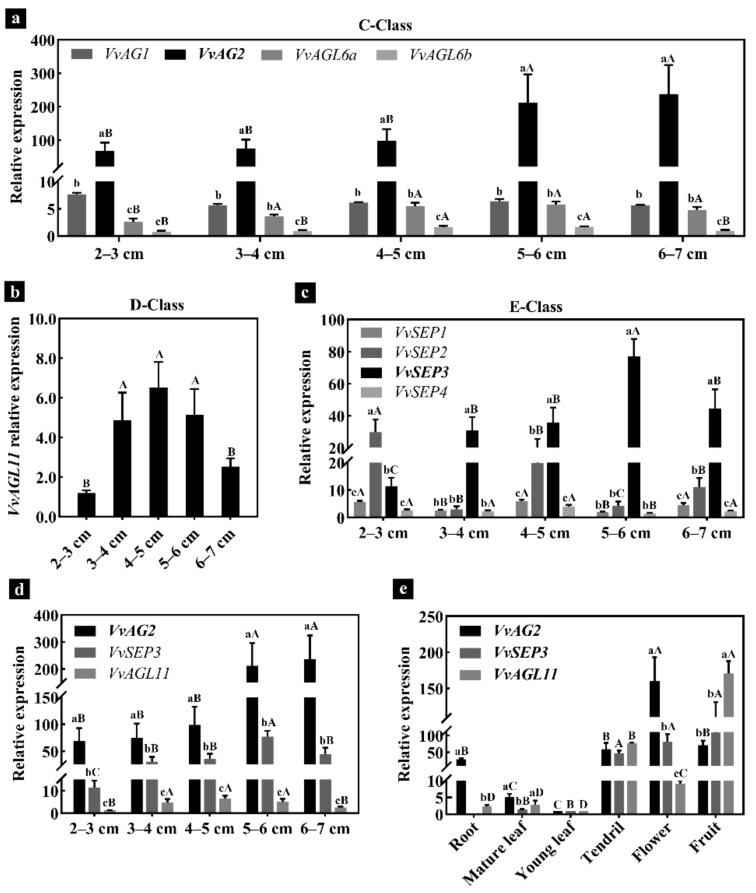
The quantitative reverse transcription PCR (qRT-PCR) analysis of genes related to ovule formation. (**a**) Fluorescence quantitative analysis of C-Class MADS-box genes in inflorescences of different lengths. (**b**) Expression of D-Class MADS-box genes in grapes. (**c**) The expression of E-Class MADS-box genes. (**d**) Fluorescence quantitative analysis of VvAG2, VvSEP3 and VvAGL11 related to ovule formation of ‘Xiangfei’ grapes. (**e**) Tissue differential expression. Capital letters represent the comparison of the expression differences of a gene at various stages or tissues (*p* < 0.05), lowercase letters represent the comparison of different genes at various stages or tissues (*p* < 0.05). Error bars indicate standard errors. Values are mean ± standard errors.

**Figure 5:**
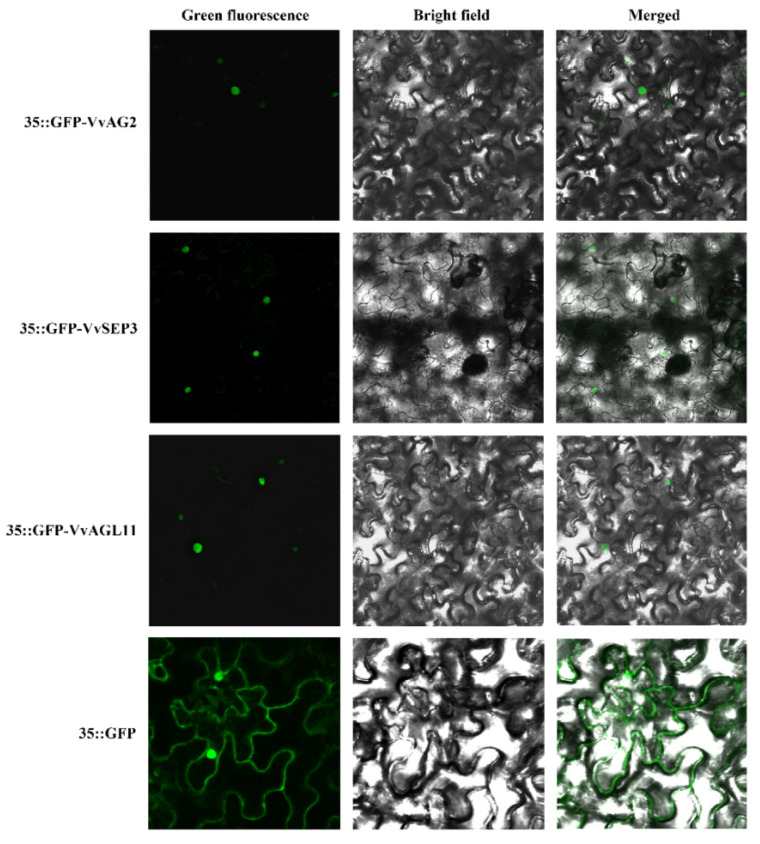
Subcellular localization of the VvAG2, VvSEP3 and VvSEP3 proteins in *N. benthamiana*. GFP-AG2, GFP-SEP3, GFP-AGL11 are located in the nucleus of *Nicotiana benthamiana*, GFP alone is located in the whole cell. Fluorescence (the left column), bright field (the middle column) and combined images (the right column) were obtained by using a confocal microscope after 48 h of *Agrobacterium* infiltration.

According to the classification of MADS-box gene families in grapes, we analyzed the expression of C-class, D-class and E-class genes associated with ovule formation. The materials used for analysis were inflorescences before and after the ovule primordium formation period (the inflorescence length was 4–5 cm). The quantitative reverse transcription PCR (qRT-PCR) analysis revealed that the expression of C class gene *VvAG2* (*VvAGAMOUS*) was significantly higher than that of *VvAG1* (*VvMADS1*), *VvAGL6a* (*VvMADS3*), and *VvAGL6b* (*VvMADS6*) genes at all stages when the inflorescence length was 2–7 cm (Figure 4a). The *VvAGL11* (*VvMADS5*) gene belonging to D-Class had a higher expression level when the inflorescence length was 3–6 cm, and its expression level reached the highest level when the inflorescence length was 4–5 cm. However, the expression of VvAGL11 gradually decreased in 5–6 cm and 6–7 cm long inflorescences (Figure 4b). In the E-Class, the expression levels of *VvSEP3* (*VvMADS4*) in inflorescence lengths of 3–4, 4–5, 5–6 and 6–7 cm were significantly higher than those of *VvSEP1* (*VvMADS2*), *VvSEP2* and *VvSEP4* (Figure 4c). Analyzing the relative expression levels of *VvAG2*, *VvSEP3* and *VvAGL11* genes during the ovule formation of ‘Xiangfei’ grapes, it was found that the expression of *VvAG2* was significantly higher than that of *VvSEP3* and *VvAGL11* (Figure 4d). We further analyzed the expression of *VvAG2*, *VvSEP3* and *VvAGL11* genes in different tissues of ‘Xiangfei’ grapes. The results showed that the expression of *VvAG2* in flowers was the highest, while the expression in roots, mature leaves and young leaves was low, and there were also certain expressions in tendrils and fruits. The highest expression levels of *VvSEP3* and *VvAGL11* were detected in fruits (Figure 4e). Furthermore, the subcellular localization results of VvAG2, VvSEP3 and VvAGL11 revealed that they all localized to the nucleus (Figure 5), which was consistent with their known function as transcription factors.

**Figure 6:**
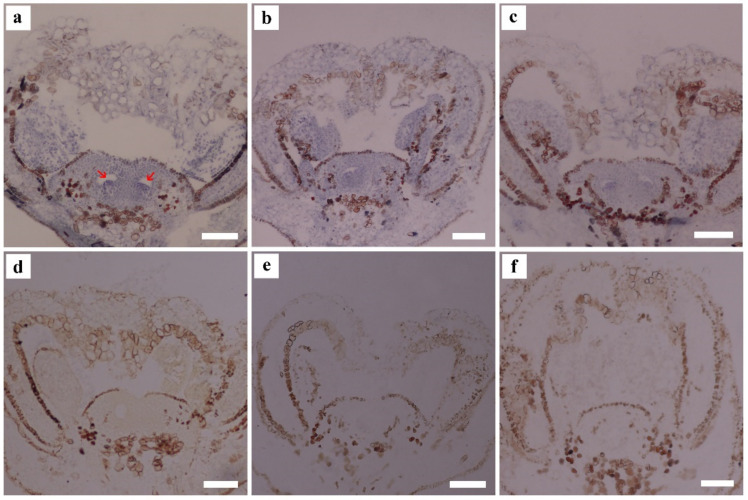
In situ hybridization of *VvAG2*, *VvSEP3* and *VvAGL11* at the stage of ovule primordium formation. (**a**) In situ hybridization of *VvAG2*. (**b**) The hybridization signal of *VvSEP3*. (**c**) In situ hybridization of *VvAGL11*. The negative control of *VvAG2* (**d**), *VvSEP3* (**e**) and *VvAGL11* (**f**). Red arrows indicate a hybridization signal. Inflorescence length: 4–5 cm. Scale bars = 200 μm.

In situ hybridization experiments were performed on ‘Xiangfei’ grape inflorescences at the stage of ovule primordium formation. In the 4–5 cm inflorescences, *VvAG2* was expressed at carpel and ovule primordia and a strong hybridization signal was observed at the ovule primordium (Figure 6a). The *VvSEP3* gene was found in the stamen, carpel and ovule primordium, and the hybridization signal was weaker than that of *VvAG2* (Figure 6b). A weak *VvAGL11* gene signal can be observed in the floral organ primordium (Figure 6c). 

### 3.4. Overexpression of VvAG2 Caused the Increment of Ovule Numbers and Early Flowering in Micro-Tom Tomato

**Figure 7:**
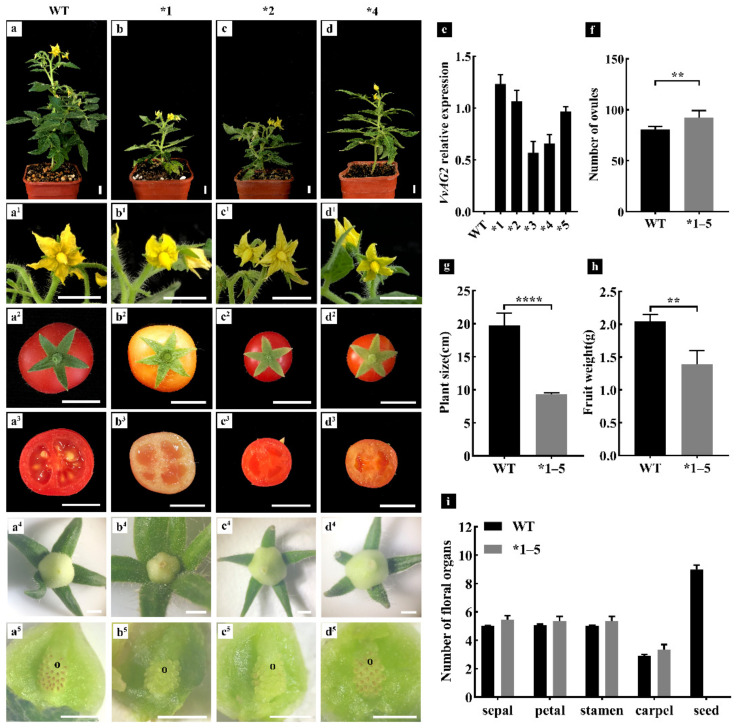
Functional analysis of transgenic tomato plants expressing *VvAG2*. (**a**,**a^1^**–**a^3^**) Wild-type Micro-Tom plant, flower and fruit organs. Scale bars = 1 cm. (**b**–**d**) Independent transgenic lines expressing *VvAG2* plant. (**b^1^**–**b^3^**, **c^1^**–**c^3^**, **d^1^**–**d^3^**) Flower and fruit organs of transgenic lines, scale bars = 1 cm. (**a^5^**,**b^5^**,**c^5^**,**d^5^**) Ovules in one locule of young fruit (**a^4^**,**b^4^**,**c^4^**,**d^4^**, one week after anthesis), scale bars = 1 mm. (**e**) *VvAG2* relative expression of wild-type and transgenic plants *1–5. (**f**–**h**) Ovules number, plant size and fruit weight in wild-type and transgenic plants. (**i**) The number of floral organs in wild-type and transgenic plants. o, ovule primordia. Error bars indicate standard errors. Values are mean ± standard errors. Significance analysis was conducted with two-tailed Student’s *t*-tests (** *p* < 0.01, **** *p* < 0.0001).

Since the *VvAG2* gene had the highest spatiotemporal expression level during the ovule formation of ‘Xiangfei’ grapes, its function on ovule development has drawn our attention. The *VvAG2* gene cloned from the ‘Xiangfei’ grape was transferred into Micro-Tom tomato plants by constructing the constitutive expression vector *35S::VvAG2*. Five independent transgenic lines (Figure 7e, *1–5) were obtained. Due to a large number of ovules in each locule of tomato and the rapid development rate, we observed and counted the number of ovules (Figure 7(a^5^–d^5^)) in young fruits (Figure 7(a^4^–d^4^)) one week after anthesis using a stereo microscope. The average number of ovules per locule was 27.73 and the average number of ovules in the small fruit of the transgenic tomato lines was 92.33, which was higher than the average number of ovules of the wild type (80.69) (Figure 7f). The average carpel number of transgenic lines was 3.33, while the average carpel number of the wild-type tomato was 2.91 (Figure 7i). Meanwhile, the number of floral organs (sepals, petals, and stamens) of transgenic tomatoes was slightly higher than that of the wild type (Figure 7i). The transgenic lines flowered earlier than the wild-type plants and were shorter in height (Figure 7a–d,g). No seeds were formed in the fruits of the transgenic lines (Figure 7i), and the single fruit weight was significantly lower than that of the wild type (Figure 7h).

### 3.5. VvAG2 Regulates Ovule Formation Together with VvSEP3 and VvAGL11

**Figure 8:**
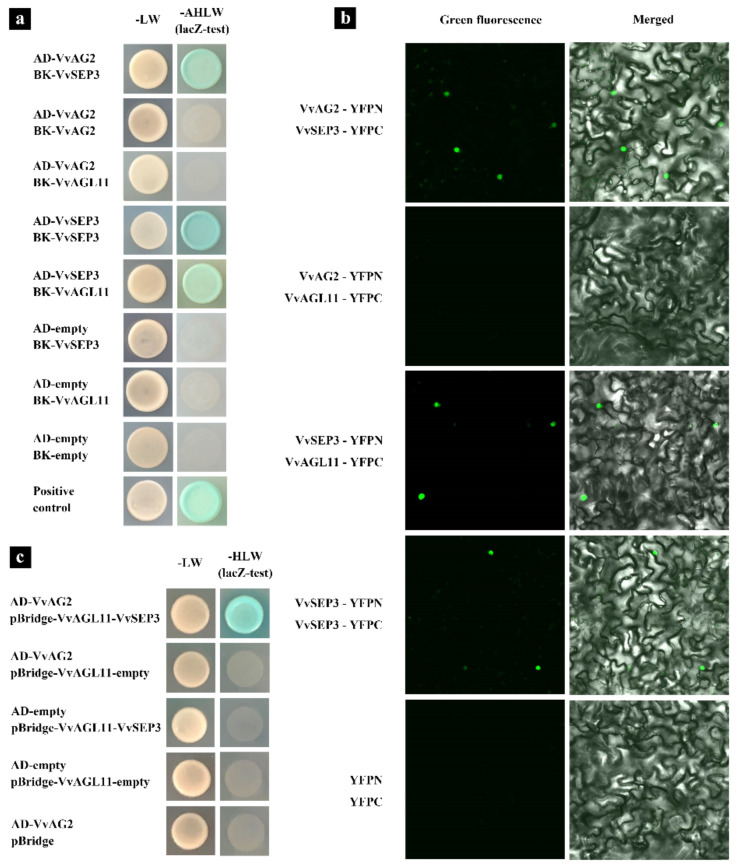
The interaction between MADS-box family proteins VvAG2, VvSEP3 and VvAGL11. (**a**) Y2H assay showed that both VvAG2 and VvAGL11 interacted with VvSEP3, and VvSEP3 could interact with itself. PGBKT7-53 (p53) and PGADT7-T (SV40 large T-antigen) were used as a positive control. (**b**) BiFC experimental analysis verified the interaction of VvAG2, VvSEP3 and VvAGL11. (**c**) Y3H assay showed that VvAG2 and VvAGL11 could interact when VvSEP3 was produced.

Since the transgenic tomato plants expressing *VvAG2* produced a higher ovule number than the wild type and the protein of MADS-box family usually need to form tetramer complexes during the formation of floral organs, the interactions between MADS-box proteins VvAG2, VvSEP3 and VvAGL11 in the grapevine ‘Xiangfei’ were analyzed. The yeast two-hybrid results showed that VvAG2 itself cannot form a homodimer, nor does it interact with VvAGL11. For VvSEP3, it can not only form a homodimer, but also interact with both VvAG2 and VvAGL11. VvAGL11, which was similar to VvAG2, cannot form a homodimer on its own or interact with VvAG2 (Figure 8a). The results of the bimolecular fluorescence complementation test were consistent with those of the yeast two-hybrid test (Figure 8b). 

Due to the limitation of Yeast Two-Hybrid (Y2H), which can only detect the interaction of two proteins, the yeast three-hybrid assay was employed for evaluating the interactions of VvAG2, VvSEP3 and VvAGL11 (Figure 8c). When VvAG2, VvSEP3, VvAGL11 were simultaneously expressed, the yeast strains grew well on the selected medium. In the absence of VvSEP3, the yeast strains could not grow on the medium, which indicated that there was no direct interaction between VvAG2 and VvAGL11. These results demonstrated that the interaction between VvAG2 and VvAGL11 was linked through VvSEP3. When VvSEP3 and VvAGL11 were constructed into the same vector, there was no interaction between VvSEP3 and VvAGL11 without the AD vector. The results of this experiment illustrates that no self-activation phenomenon occurs when VvSEP3 and VvAGL11 are constructed on the same vector.

## 4. Discussion

### 4.1. The Ovule Formation of Grapevine Cultivar ‘Xiangfei’ Is Similar to Arabidopsis

The ovule is the precursor to the seed and is located in the fourth wheel floral organ. It is an important part of the pistil and starts from the placental meristem inside the carpel. Most varieties of grapes have two carpels and four ovules. In this study, the number of carpels and ovules of ‘Xiangfei’ grapes were counted. We found that there is a phenomenon of multi-carpels with 2–5 carpels in the ‘Xiangfei’ grape, which contained more ovules. Formation of the ovule primordia occurs in the 4–5 cm inflorescences, followed by the funiculus and integument. With the completion of ovule inversion, the embryo sac begins to develop. The formation and development pattern of the grapevine ‘Xiangfei’ is very similar to that of *Arabidopsis thaliana*, both of which have undergone the formation and development of the ovule primordium, the inversion stage of ovule, and the formation and development stage of the female gametophyte [30].

### 4.2. The Ovule Formation of ‘Xiangfei’ Grape Is Regulated by MADS-box Family Genes

MADS-box genes play an extremely important role in the regulation of floral organ development in plants [31]. The flower organ development model regulated by MADS-box genes in *A. thaliana* is the ABCDE (Floral organ characteristics in *Arabidopsis thaliana* are regulated by A, B, C, D, and E class floral homeotic genes) model [32]; C, D and E function genes are considered to regulate ovule formation and development [33]. MADS-box genes in grapes can also be classified into A, B, C, D and E by reference to grape genome sequencing and classification in *A. thaliana* [32,34]. In this study, we analyzed the expression of C, D and E genes during the ovule formation stage of ‘Xiangfei’ grapes. C-Class gene *VvAG2*, D-Class gene *VvAGL11* and E-Class gene *VvSEP3* may play a role in the appearance of the ovule primordium. Our findings were consistent with studies in grapevine cultivars ‘Cabernet Sauvignon’ and ‘Pinot Noir’ [32,35].

### 4.3. Transgenic Tomato ‘Micro-Tom’ Plants Expressing VvAG2 Produced More Ovules and Other Floral Organs

The C function gene *AGAMOUS* (*AG*) is involved in the formation of the stamen, carpel, and ovule primordium [36,37,38,39,40]. The function loss of the *AG* gene results in the mutant flowers containing only sepals and petals [41]. The *TAG1* and *TAGL1* genes belonging to C-class MADS-box genes in the tomato are also involved in the formation of flower organs. When the *TAG1* gene is inhibited, the stamen of the tomato turns into petal-like organs, and the development of carpel is also abnormal [42,43]. *TAGL1* over-expression experiments demonstrated that the sepal organs changed into carpel and developed into fleshy fruit tissue, while the silenced *TAGL1* gene led to malformations of seed development [44]. In this study, the *VvAG2* gene was highly expressed during flower organ formation and development, and the number of carpel and ovule was significantly increased when the *VvAG2* gene was transferred into the Micro-Tom tomato. This suggests that the *VvAG2* gene may be involved in ovule formation.

Interestingly, we found no seeds in the fruits of all transgenic lines (Figure 7(b^3^–d^3^,i)). We observed the morphological dissection of the flower buds from wild-type Micro-Tom and *VvAG2* transgenic lines through paraffin sections. It can be clearly found that the stamen primordium of *VvAG2* transgenic lines showed different degrees of developmental defects compared with the wild type (Supplementary Figure S1), thus we speculated that the heterologous expression of *VvAG2* might affect the normal development of stamens in tomatoes, resulting in the inability to produce normal fertile pollen in transgenic plants. This speculation needs further experimental verification.

### 4.4. VvAG2, VvSEP3 and VvAGL11 Proteins of MADS-box Family Participate in the Formation of Grape Ovules by Forming the Tetrameric Complex

Tetramerization of MADS-domain transcription factors is one of the major determinants controlling the formation of the different floral organ types and plays a central role in the evolution of higher plants [45,46]. It has been confirmed in Arabidopsis, tomato, rice (*Oryza sativa*) and other plants that the MADS-box family transcription factors can form multimers to regulate the development of floral organs [47,48,49]. The AG/AG–SEP/SEP tetramer of Arabidopsis regulates the development of the carpel, while the AG/SEP–STK/SEP tetramer regulates the development of the ovule [50]. In addition, SEPALLATA3 (SEP3) plays a pivotal role in mediating multimerization [15,46].

**Figure 9:**
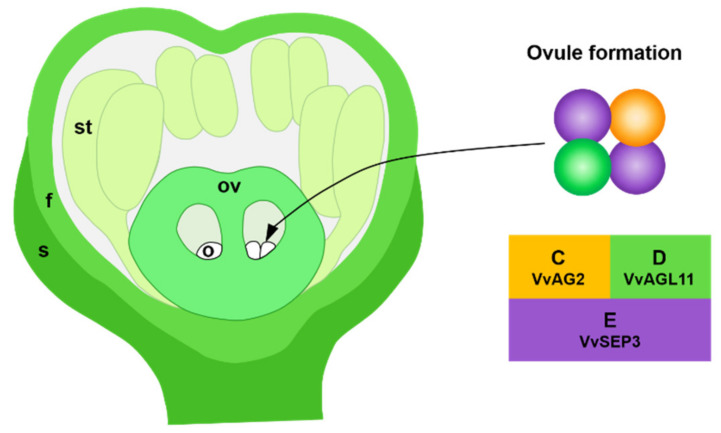
Diagram: VvAG2/VvSEP3 and VvAGL11/VvSEP3 form tetramers and participate in the formation of ovules. The orange sphere represents VvAG2 protein; the purple sphere represents VvSEP3; the green sphere represents VvAGL11. o, ovule primordia; s, sepal primordia; f, flower cap primordia; st, stamen primordia; ov, ovary.

In this study, we observed that VvAG2 and VvAGL11 could not form a homodimer on their own, nor could they interact with each other, but they both had an interaction relationship with VvSEP3. However, VvSEP3 can form a homodimer and interact with VvAG2 and VvAGL11. These results indicate that VvAG2 and VvAGL11 can form tetramers by interacting with VvSEP3, where VvSEP3 acts as a bridge in the tetramer. In summary, we believe that the grape MADS-box family transcription factor complexes VvAG2/VvSEP3 and VvAGL11/VvSEP3 form tetramers that may be involved in the formation of ovules (Figure 9).

## 5. Conclusions

The current study analyzed the spatiotemporal expressions of MADS-box genes *VvAG2*, *VvSEP3* and *VvAGL11* in the formation of ovule primordium in ‘Xiangfei’ grape, and found the expression of *VvAG2* was highest. The *VvAG2* gene was transferred into Micro-Tom tomato, which resulted in an increase in the number of ovules. VvAG2, VvSEP3 and VvAGL11 proteins can form tetramers. Our findings indicated that *VvAG2*, *VvSEP3* and *VvAGL11* are involved in the formation of ovule in ‘Xiangfei’ grape.

## Data Availability

All relevant data are included in the manuscript and Supplementary Files.

## Supplementary Materials

The following are available online at https://www.mdpi.com/article/10.3390/genes12050647/s1, Figure S1: Anatomical structure of flower buds from wild-type Micro-Tom and transgenic lines of VvAG2 using Safranin O-Fast Green staining. Table S1: List of primers used in this study. Table S2: List of sequence names and accession numbers of all the genes involved in this study.
