# Supplementary material for: MADS-Box Protein Complex VvAG2, VvSEP3 and VvAGL11 Regulates the Formation of Ovules in Vitis vinifera L. cv. ‘Xiangfei’
Source: Genes (Basel). 2021 Apr 26;12(5):647. doi: 10.3390/genes12050647 (PMC8146481; doi:10.3390/genes12050647)
Supplement: Supplementary file 1 [file genes-12-00647-s001.zip › genes-1153294-supplementary.pdf]

**Supplementary figure S1.** Anatomical structure of flower buds from wild-type Micro-Tom and transgenic lines of *VvAG2* using Safranin O-Fast Green staining. (a) Cross section of flower buds from wild-type Micro-Tom. (b-d) Cross section of flower buds from transgenic lines. Scale bars = 200 μm. Bud length = 3–5 mm. Green circle indicate the stamen primordium. (e) The various stages of the development of flower buds into rip fruit in Micro-Tom. Scale bars = 1 cm.

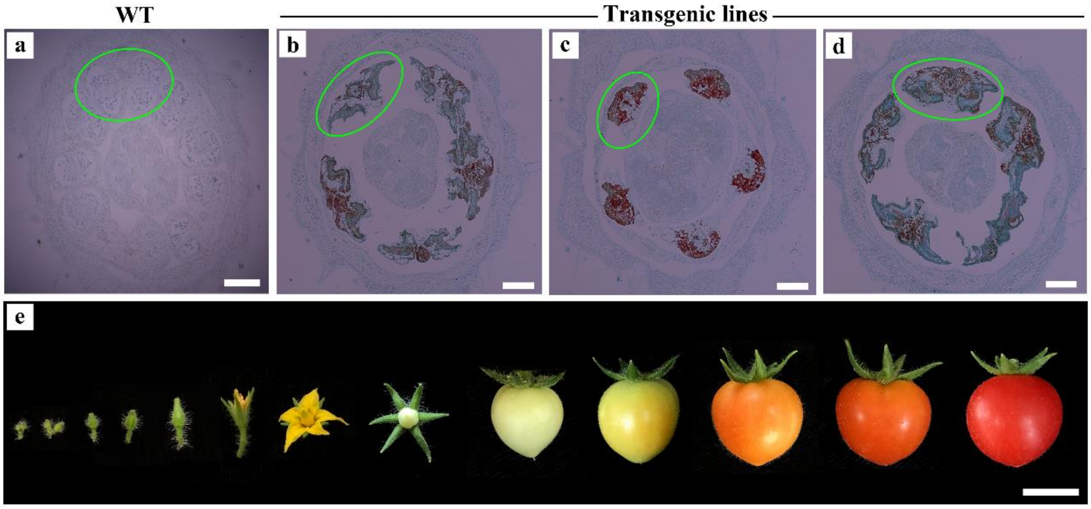


**Supplementary table S1.** List of primers used in this study.

| Gene | Primer sequence (5′→3′) |
|---|---|
| **Primers of qRT-PCR analysis** | |
| *VvAG1* | F: TCTGGGTGAGGCTCTTAG |
| | R: TGTGCTCGCAGAAATAAG |
| *VvAG2* | F: GTCCGTTTCTGAAGCCAAT |
| | R: CCCTGATTCTACTTATGCCTTT |
| *VvAGL6a* | F: CTGCGAACCCGAACCCAT |
| | R: AACCCACCCTTGGATGAA |
| *VvAGL6b* | F: CTTTCTGTGCTCTGCGATGC |
| | R: CGGTATCGCTCAATGGTT |
| *VvAGL11* | F: ATGGGTGATTCCTTGGCTT |
| | R: CGAGAAACTAATGCCTGGATG |
| *VvSEP1* | F: GCCCTTCAACGAACTCAG |
| | R: CATCCAGCTTCCTCGTCA |
| *VvSEP2* | F: AGCCGTGGCAAACTCTAT |
| | R: GTTTAGCGGGTCCAAGTC |
| *VvSEP3* | F: CTATGGTCGGCAACAAGC |
| | R: ACACTGGGGCCTGCTGCT |
| *VvSEP4* | F: GCCGCACATAATCTACCA |
| | R: TCAACCCGCAGCACAGGA |
| *UBQ* | F: GCTCGCTGTTTTGCAGTTCTAC |
| | R: AACATAGGTGAGGCCGCACTT |
| **Primers of transgenic vector and Subcellular localization (*35S::GFP-gene*)** | |

| | |
|---|---|
| *VvAG2* | F: GGACTAGTATGGGAAGGGGGAAGATC |
| | R: GGGTTACCCACTAATTGAAGAGCT |
| *VvSEP3* | F: GGAAGATCTATGGGGAGAGGTAGGGTTGA |
| | R: GGACTAGTTGGCAACCATCCCGGCATGTAG |
| *VvAGL11* | F: GGAAGATCTATGCTTTGTGTAGTGAACATGGGGA |
| | R: GGACTAGTCCCGAGATGGAGGACCTTCTTA |
| **Primers of *in situ* hybridization** | |
| *VvAG2* | F: TAATACGACTCACTATAGGGAGAGCCTGGAGATTCGG |
| | R: AATTAACCCTCACTAAAGGGCATTCTCGGCTATCCTTGC |
| *VvSEP3* | F: TAATACGACTCACTATAGGGCATGCTGGATCAACTCACC |
| | R: AATTAACCCTCACTAAAGGGCTTGTTGCCGACCATAGC |
| *VvAGL11* | F: TAATACGACTCACTATAGGGATGGGTGATTCCTTGGCTT |
| | R: AATTAACCCTCACTAAAGGGCGAGAAACTAATGCCTGGATG |
| **Primers of Y2H** | |
| *AD-VvAG2* | F: CGACGTACCAGATTACGCTCATATGATGGGAAGGGGGAAGATC |
| | R: ATCTACGATTCATCTGCAGCTCGAGCACTAATTGAAGAGCT |
| *AD-VvSEP3* | F: CGACGTACCAGATTACGCTCATATGATGGGGAGAGGTAGGGTTGA |
| | R: ATCTACGATTCATCTGCAGCTCGAGTGGCAACCATCCCGGCATGTAG |
| *AD-VvAGL11* | F: CGACGTACCAGATTACGCTCATATGATGCTTTGTGTAGTGAACATGGGGA |
| | R: ATCTACGATTCATCTGCAGCTCGAGCCCGAGATGGAGGACCTTCTTA |
| *BK-VvAG2* | F: GATCTCAGAGGAGGACCTGCATATGATGGGAAGGGGGAAGATC |
| | R: CGACGGATCCCCGGGAATTCCTCACTAATTGAAGAGCT |
| *BK-VvSEP3* | F: GATCTCAGAGGAGGACCTGCATATGATGGGGAGAGGTAGGGTTGA |
| | R: CGACGGATCCCCGGGAATTCCTTGGCAACCATCCCGGCATGTAG |
| *BK-VvAGL11* | F: GATCTCAGAGGAGGACCTGCATATGATGCTTTGTGTAGTGAACATGGGGA |
| | R: CGACGGATCCCCGGGAATTCCTCCCGAGATGGAGGACCTTCTTA |
| **Primers of Y3H** | |
| *pBridge-VvAGL11* | F: ACAGTTGACTGTATCGCCGGAATTCATGCTTTGTGTAGTGAACATGGGGA |
| | R: AGCTTGGCTGCAGGTCGACGGATCCCCCGAGATGGAGGACCTTCTTA |
| *pBridge-VvSEP3* | F: AGAAGAAGAGAAAGGTGGCGGCCGCATGGGGAGAGGTAGGGTTGA |
| | R: ACATGGGAGATCAGCCCGAAGATCTTGGCAACCATCCCGGCATGTAG |
| **Primers of BiFC** | |
| *SPYCE-VvAG2* | F: ACACGGGGGACTCTAGAGTTATGGGAAGGGGGAAGATC |
| | R: AGGCCTGAGCCCGGTTCACTAATTGAAGAGCT |
| *SPYCE-VvSEP3* | F: ACACGGGGGACTCTAGAGTTATGGGGAGAGGTAGGGTTGA |
| | R: AGGCCTGAGCCCGGTTTGGCAACCATCCCGGCATGTAG |
| *SPYCE-VvAGL11* | F: ACACGGGGGACTCTAGAGTTATGCTTTGTGTAGTGAACATGGGGA |
| | R: AGGCCTGAGCCCGGTTCCCGAGATGGAGGACCTTCTTA |
| *SPYNE-VvAG2* | F: AAACGGGGGACTCTAGAGTTATGGGAAGGGGGAAGATC |
| | R: AGGCCTGAGCCCGGTTCACTAATTGAAGAGCT |
| *SPYNE-VvSEP3* | F: AAACGGGGGACTCTAGAGTTATGGGGAGAGGTAGGGTTGA |
| | R: AGGCCTGAGCCCGGTTTGGCAACCATCCCGGCATGTAG |
| *SPYNE-VvAGL11* | F: AAACGGGGGACTCTAGAGTTATGCTTTGTGTAGTGAACATGGGGA |
| | R: AGGCCTGAGCCCGGTTCCCGAGATGGAGGACCTTCTTA |

**Supplementary table S2.** List of sequence names and accession numbers of all the genes involved in this study.

| **Gene** | **Gene ID** | **Alternative Names** | **Location** |
|---|---|---|---|
| *VvAG1* | LOC100232864 | VIT_12s0142g00360 | chr12 |
| *VvAG2* | LOC100261787 | VIT_10s0003g02070 | chr10 |
| *VvAGL6a* | LOC100232868 | VIT_15s0048g01270 | chr15 |
| *VvAGL6b* | LOC100256085 | VIT_16s0022g02330 | chr16 |
| *VvAGL11* | LOC100232870 | VIT_18s0041g01880 | chr18 |
| *VvSEP1* | LOC100232867 | VIT_14s0083g01050 | chr14 |
| *VvSEP2* | LOC100251943 | VIT_17s0000g05000 | chr17 |
| *VvSEP3* | LOC100232869 | VIT_01s0010g03900 | chr01 |
| *VvSEP4* | LOC100232936 | VIT_01s0011g00110 | chr01 |
